# mHealth Technology Translation in a Limited Resources Community—Process, Challenges, and Lessons Learned From a Limited Resources Community of Chiang Mai Province, Thailand

**DOI:** 10.1109/JTEHM.2021.3055069

**Published:** 2021-01-27

**Authors:** Waraporn Boonchieng, Jintana Chaiwan, Bijaya Shrestha, Manash Shrestha, Adam J.O. Dede, Ekkarat Boonchieng

**Affiliations:** 1 Faculty of Public HealthChiang Mai University 26682 Chiang Mai50200Thailand; 2 Faculty of Social Sciences and HumanitiesMahidol University 26685 Nakhon Pathom73170Thailand; 3 Unit of Excellence on Clinical Outcomes Research and Integration, School of Pharmaceutical SciencesUniversity of Phayao 90440 Mae Ka56000Thailand; 4 Data Science Research CenterDepartment of Computer ScienceFaculty of Science, Chiang Mai University 26682 Chiang Mai50200Thailand

**Keywords:** Healthcare deliveries, community-based distribution, mhealth, technology translation, geographic information system, public health

## Abstract

This report aims to provide practical advice about the implementation of a public health monitoring system using both geographic information system technology and mobile health, a term used for healthcare delivery via mobile devices. application amongst household residents and community stakeholders in the limited resource community. A public health monitoring system was implemented in a semi-rural district in Thailand. The challenges encountered during implementation were documented qualitatively in a series of monthly focus group discussions, several community hearings, and many targeted interviews. In addition, lessons learned from the expansion of the program to 75 other districts throughout Thailand were also considered. All challenges proved solvable yielding several key pieces of advice for future project implementation teams. Specifically, communication between team members, anticipating technological challenges, and involvement of community members are critical. The problems encountered in our project were mainly related to the capabilities of the data collectors and technical issues of mobile devices, internet coverage, and the GIS application itself. During the implementation phase, progressive changes needed to be made to the system promptly, in parallel with community team building in order to get the highest public health impact.

## I. Introduction and Clinical Need

Geographic information systems (GIS) are computer systems designed to capture, store, manipulate, analyze, manage, and present spatial and geographical data [1]. Globally, GIS technology has great potential to aid public health efforts and has been applied to public health in at least three ways. First, GIS technology can increase understanding of how and where health care services are accessed, leading to more targeted plans to address coverage and access issues [2], [3]. Second, interactive maps produced using GIS techniques are useful for monitoring the live spread of diseases in the population by strengthening disease surveillance, examining disease risk factors, and planning and evaluating interventions [4], [5]. Third, GIS can also aid the development of community profiles, giving healthcare workers knowledge of demographic, economic, healthcare, and lifestyle characteristics of the population as well as information about exposure to potential environmental hazards [2], [6]–[8].

Combining GIS with mobile health (mHealth) initiatives can lead to powerful benefits. mHealth is any use of mobile phones as a platform for delivery of health care, health monitoring, or health data collection. Example applications can range from simple reminders prompting patients to take medications on time to diagnostic questionnaires. mHealth is fast and cheap while maintaining patient privacy, and due to the ease of collecting geographic data from mobile devices, mHealth can be easily integrated with GIS allowing for powerful public health inferences. A recent review found that most research employing mobile technologies and GIS for enhancing health care and health information systems report positive results, but almost none discuss implementation issues [9]. Lack of practical implementation descriptions may limit widespread adoption of these systems.

In 2014, an interdisciplinary research team from Chiang Mai University, addressed these issues and developed first ever community healthcare profiling system using mHealth technology [10]. This system allowed trained data collectors to gather data from community members and upload that data directly into a cloud-based database. For this project, the information collected included demographics, disabilities, disease prevalence, psychological disorders, drug abuse, community activities, and beliefs (See Table 1 and ref. 20 for more detail). This project focused on the semi-rural district of Saraphi in Chiang Mai province and aimed to give healthcare providers in Chiang Mai geographically indexed and easily searchable information to profile the public health of their communities using an up-to-date digital format. Since our original report [10], the project has scaled up from 1 province to 52 provinces over the past five years.

**TABLE 1:** Data Attributes Being Collected in the GIS-Linked Health Information System at Saraphi District

Parameters	Attributes
Demographic and general information	a. Age b. Sex c. Occupation d. Income e. Education f. Medical welfare g. Role and position in the community h. Folk wisdom i. Special care needs j. Work experience
Health status	a. Chronic diseases b. Infectious diseases c. Mental health d. Disability
Health-related behaviors	a. Substance abuse b. Eating behaviors c. Medication adherence d. Exercise
Environmental health awareness	a. Waste management b. Vector-borne diseases c. Wastewater treatment d. Sanitation e. Household safety f. Pets and animal disturbance
Access to health services	a. Self-care b. Primary healthcare c. Choice of health service system d. Primary care service development needs e. Primary care service satisfaction

Indeed, there has been a sudden surge in publications of studies using GIS to address public health issues throughout Thailand. Investigations have focused on the incidence and spatial patterns of major infectious diseases such as malaria [11]–[13], dengue [14]–[16], and tuberculosis [17]. Some studies have also utilized GIS to identify areas and populations at risk of chronic diseases such as cancer and hypertension [18], [19]. In one study, Rujirakul *et al.* studied the distribution and risk factors of parasitic liver fluke infections in Surin Province in Eastern Thailand using GIS-based software for proper allocation of prevention and control measures [20].

An important reason for this success is that Thailand has sufficient smartphone penetration to make mHealth implementation feasible even in rural locations. 63% of Thais have smartphones, compared to 46% across South East Asia [21]. And even in rural locations, the cheapest handsets and data plans are now powerful enough to support GIS-based mHealth applications.

In light of the public health need presented by the current COVID-19 pandemic, and the increasing worldwide penetration of smartphone ownership, we believe that successes like those accomplished in Thailand can be accomplished elsewhere. To address a lack of practical advice on mHealth and GIS public health system implementation, this paper will share an updated review of our experiences implementing Thailand’s first ever community healthcare profiling system [10]. To highlight the type of insight made possible through the collection of this type of dataset, we will share an example analysis of dengue fever prevalence. However, our main focus will be on the aspects of our experience that have proved most useful as the project has been expanded to 51 more provinces over the last five years. Our goal is to provide teams seeking to implement similar systems with an account of the challenges that we faced and the solutions we found. The primary data for this report were collected from focus groups involving the project implementation team members and the community stakeholders.

## II. Methods

### A. Application and Database Management

The mHealth application, “CMDigiHealth”, was previously developed as a mobile information management application, supporting both iOS and Android platforms. The application is described in detail in our previous report [10]. Briefly, the application served as a platform to distribute health surveys quickly and easily to large groups. The application also collected global positioning system (GPS) coordinates. The application’s graphic user interface was developed using HTML5 and PHP with MySQL as a database management system (DBMS). The data were stored in a private cloud server, OpenNode GCP, which operates on CentOS version 6.5 and can be made available at http://cmhealthinformatics.com/.

To ensure patient privacy, the database could only be accessed from the IP address of the Hospital of Saraphi and access also required authorized username and password information. In addition, data access was organized such that only the project director, hospital director, and head of the provincial office of Chiang Mai could access the full dataset. Access to subsets of the data was made available to individual healthcare providers as needed.

CMDigiHealth manages and queries information using GPS coordinates as its index. Using location coordinates to index files yielded high quality spatial information which served as an automatic organizing principle for storing, updating, analyzing, and visualizing the data. This meant that there was no need for any data cleaning, entry, or organizing between data collection and data availability for analysis/ visualization.

### B. Data Collection

The primary data for this report are drawn from a series of structured focus group sessions and questionnaires involving project implementation team members and community stakeholders (described in more detail in methods section E). Project implementation took place primarily between October 2013 and October 2016. Focus group sessions were carried out throughout project implementation and have continued to the time of writing. In addition, standardized metrics for user experience with the application were collected during project implementation as described below.

### C. Participants

Focus groups included the developers and designers of the GIS application and 150 trained data collectors. We also engaged 80 government officers, 12 chairmen and 30 volunteers from village health volunteer offices and community leaders as stakeholders during the development and translation of the mHealth system to the community.

### D. Ethical Consideration

Ethical clearance for this research was obtained from the Research Ethical Committee of Faculty of Nursing, Chiang Mai University, Thailand (Record Number 104/2555). Informed consent was sought from developers, data collectors, and stakeholders. All participants were clearly informed about the voluntary nature of participation during evaluation and their right to withdraw from the interviews and focus group discussions.

### E. mHealth Translational Process

The study has been split into three translational phases including preparation, implementation, and evaluation. For all three phases, questionnaires, interviews and focus group discussion guidelines, and recoding forms were used in the study. All tools were validated by a panel of nine experts of epidemiology, public health, and GIS which are researchers and staff members from Faculty of Nursing, and Faculty of Medicine, Chiang Mai University.

#### 1) Preparation Phase

During the preparation phase, the mHealth application was developed, the data collectors were recruited and trained. In addition, a public awareness campaign sought to inform community leaders, public health authorities, village health volunteers, and community residents about the benefits of and process of mHealth public health data collection before the first launch of the mHealth application.

In this phase, the finalized questionnaires were printed and used to train the data collectors in order to ensure their ability to correctly explain health questions to community members. The application developers were also interviewed by the project leaders on a regular basis in order to monitor any issues that arose with the application.

#### 2) Implementation Phase

The trained data collectors were assigned to collect health information from their assigned areas. Data collectors went into the field to collect health information using the CMDigiHealth application from community members. During this phase, data collectors and application developers worked collaboratively to solve on-site problems as they arose. We also began formal focus group discussions to identify problems and successes identified by different team members. These were held monthly throughout the duration of the project.

#### 3) Evaluation Phase

Stakeholders’ meetings were held during this phase to evaluate the collected information. In addition, a public hearing was held to discuss ethical issues surrounding the access and use of the collected data. Focus group discussions continued during this phase. These focused on producing structured guidelines that could be applied to help in the implementation of similar projects in the future.

### F. Outcome Measurement

The health data collected during project implementation provided a large range of descriptive statistics that could be assessed over geographically defined subgroups of the community to healthcare providers. This ability had previously been unavailable. Although not the focus of the present report, to provide insight into the power of this dataset and database management system, one example analysis is provided in Fig. 2. When the Dengue cases report can be mapped with the residents which were previously request for the mosquitoes’ larvae elimination from the provincial health office, the generated report can help us to assess the effectiveness of mosquitoes’ larvae elimination process.

**FIGURE 1.**
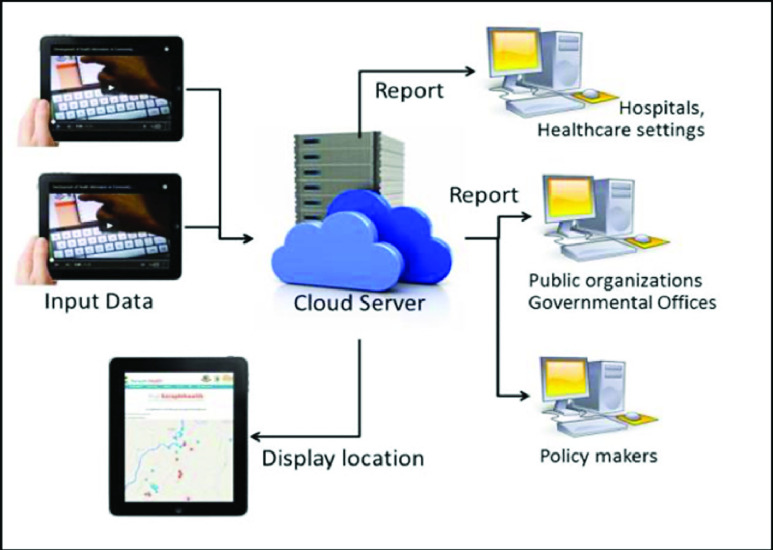
CMDigiHealth application conceptual working diagram.

**FIGURE 2.**
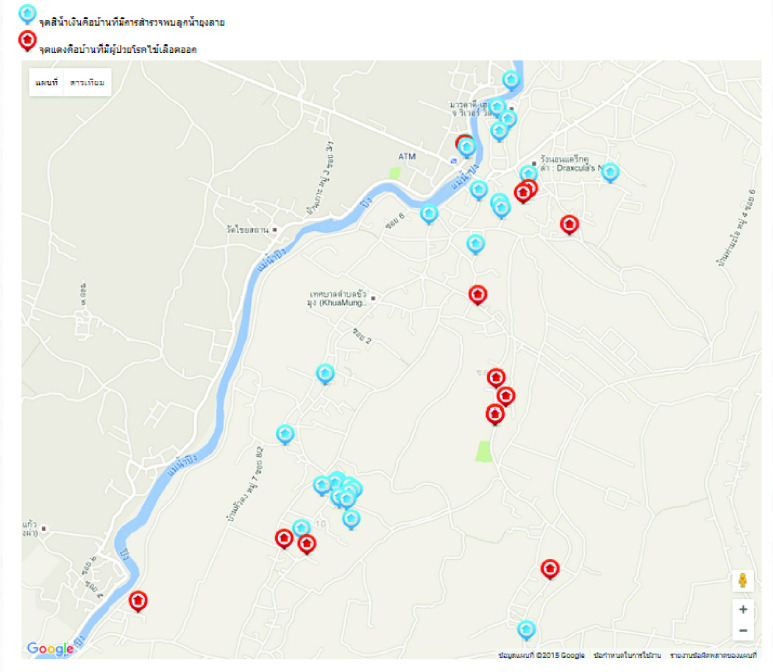
GIS-based reporting system shows the GPS location of households with dengue fever patients (red pin) and the GPS location of the natural creek/pond where mosquitoes larvae have been reported (blue pin).

To assess the usability of the application tests were performed using standards established by the International Organization for Standardization (ISO) [22]. Tests included: effectiveness, system efficiency, and user satisfaction. Effectiveness was measured by assessing the percentage of users who could complete a given set of tasks and assessed across the data collectors (n=150). System efficacy was measured by surveying the level of effort and resource use by the user to achieve usability goal(s). In addition, average time to complete each task was measured. User satisfaction tests satisfaction with the intervention, the satisfaction with the implementation process, and the satisfaction with solutions. Satisfaction tests utilized System Usability Scales (SUS) [23], [24]. SUSs are questionnaires in which participants are asked to indicate their agreement/disagreement with a series of statements on a 5-point scale. For example, SUSs used statements like, “I found this system unnecessarily complex.” and, “I thought the system was easy to use.” In addition, more general impressions of the app were gleaned from interviews with data collectors conducted throughout project implementation.

To assess the experience of project implementation including problems and solutions encountered, qualitative data from the public hearings and focus group discussions were subjected to thematic analysis. Specifically, a tacit knowledge management model was used for the focus group discussions to gather individual experiences and information. There were 4 sub-processes included: socialization, externalization, combination, and generalization which was adapted from Driedger *et al.* [25] as shown in TABLE 1. The data collectors’ satisfaction level with the solutions from the knowledge management process was measured.

To measure how easily the lessons learned from the implementation of the original program transferred to novel settings, the number of new provinces adopting and implementing the program was monitored. Finally, we assessed what factors were important in the maintenance of the program in new areas.

## III. Results

Over the study period, the health data from 53,458 (65.87% of the district) individuals and 17,069 (74.59% of the district) households were collected and validated. None of all 150 data collectors was dropped out. The collected information was made available online at http://www.cmhealthinformatics.com with user authentication. Sample of data visualization is shown in Fig. 4.

**FIGURE 3.**
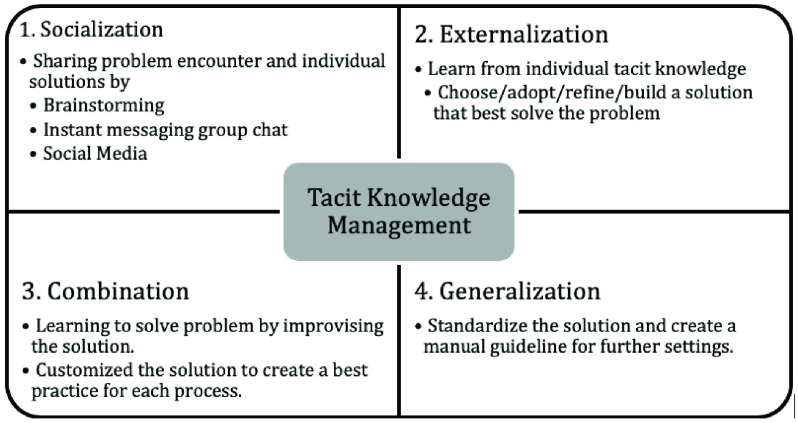
Tacit knowledge extraction model used in effectiveness assessment.

**FIGURE 4.**
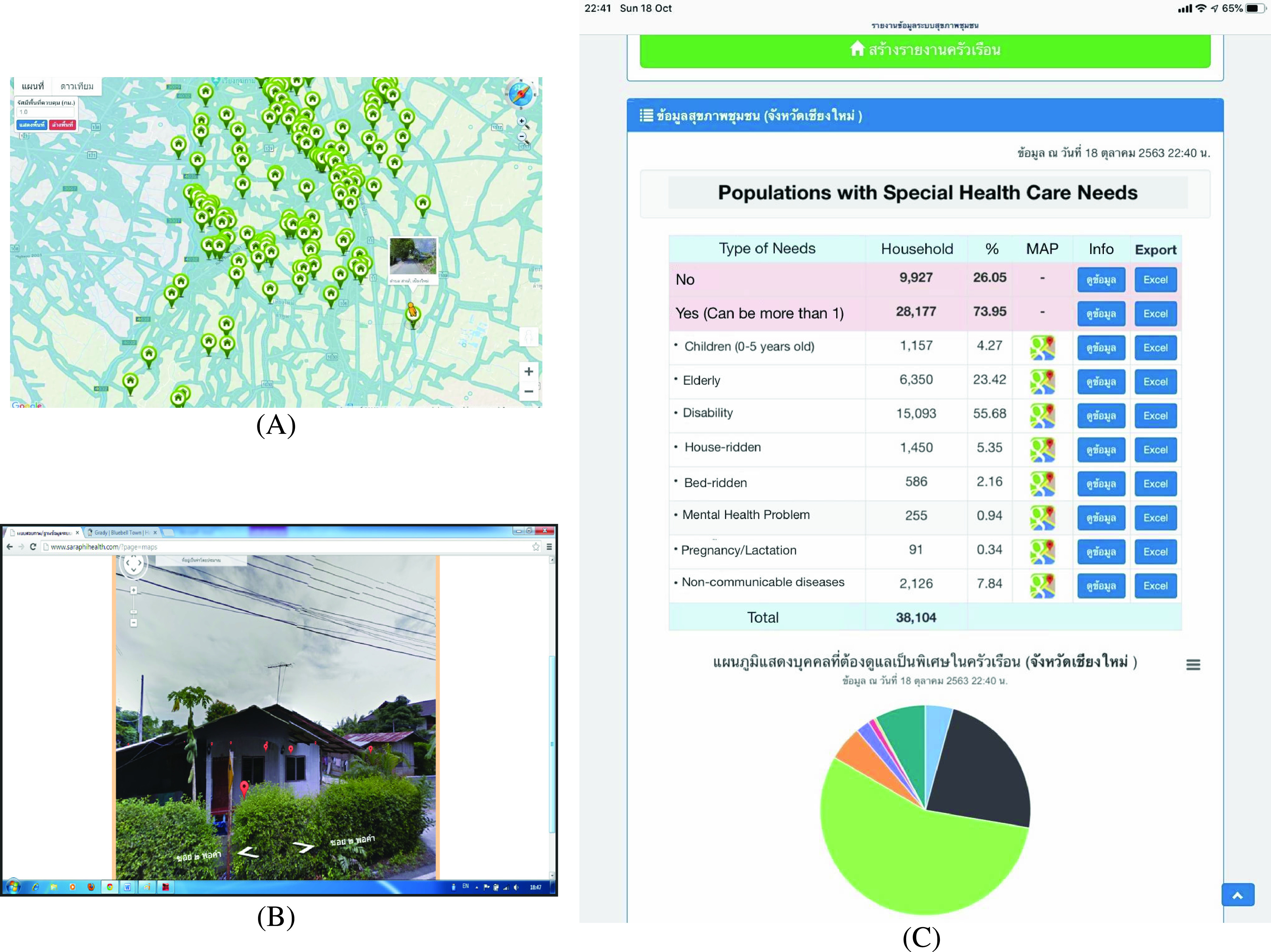
Data visualization from CMDigiHealth system shows the geographic-based data collection (A), integrated street-view (B), and descriptive statistic from each parameter and attributes collected (C).

### A. Usability of the CMDigiHealth Application

The application was developed to meet the international standard requirement for Human-centered design for interactive systems (ISO 9241-21:2019). All of our data collectors were able to complete 4 tasks given which are including (i) registration and login, (ii) complete patient health profile form, (iii) attach a photo and retrieve GPS location, and (iv) generating a report. The efficacy test found that the average time to complete each task was 13.21 (6.42 – 19.56) minutes/task. Of all 150 data collectors, 132 (88%) were able to complete all tasks with no help and 18 (12%) were able to complete all task but with minor help from the test leader. The system effectiveness found that the application was crashed 11 times in 920 launched during the tests (1.19% crashed). The SUS scores for user satisfaction with the intervention, satisfaction with the implementation process, and satisfaction with problem solutions were 87.1, 85.47, and 87.82 respectively.

Random interviews with the data collectors revealed that the final mobile application was easy to use and appropriate in design for collecting and editing data. The data collectors found it easy to record and upload data in many file types such as JPEG, PDF, and PNG.

### B. Experience of Project Implementation: Problems Encountered and Solutions Found

#### 1) Data Collectors

The majority of data collectors with experience in conducting health surveys lacked technical skills in using mobile applications for recording answers for questionnaires. For example, there were many incomplete survey forms due to the failure of the data collectors to link the completed data in one section to another section. Some data collectors were not confident submitting completed questionnaires from the field and asked the local staff at the hospital to print the interview data. They checked the accuracy and only then uploaded the data online. The data collectors also complained that the application limited the duration of data collection in the field such that they had to rush to complete the survey. These data collectors tended to be between 40 and 45 years old. Some of these experienced health volunteers did not have a smartphone, for whom we arranged to borrow mobile data collection devices from the local government authorities.

To help increase the technological fluency of our data collection team, we recruited new data collectors at secondary and vocational schools. These young data collectors possessed smartphones, had technical skills, and had free time to help with data collection. We also envisioned that utilizing youth in data collection would engage them with community problems firsthand.

Although young data collectors were internet savvy and used mobile devices fluently, many of them had difficulty understanding the sensitive nature of the information requested on the questionnaire, and they sometimes lacked the communication skills and patience needed for health research in the community. As a result, community members felt uncomfortable discussing personal issues with some of the younger data collectors.

To address these issues, we paired older experienced data collectors who lacked technical skills with younger inexperienced data collectors who had technical skills. These data collection teams proved highly effective.

#### 2) Community Residents

During the household survey, some residents did not have some information required in the questionnaire, which necessitated more than one interview to complete the survey. Some of the residents were irritated due to repeated interviews. Some topics of the questionnaire asked about sensitive issues such as household income and debts. We found that it was important to train data collectors to be sensitive when dealing with community members. In addition, we utilized a public awareness campaign so that community members would already have heard about the project before being contacted by data collectors. This campaign included video and print media (e.g. https://www.youtube.com/watch?vDop5ZZoGqIBE, https://www.youtube.com/watch?vDc-ShPUuUq2U).

#### 3) Access to Local Communities

At the start of this project, some community leaders were busy and could not envision the benefits of the project to their community. Collaboration at the community level with the leaders and local organizations was low at the beginning, and this hampered data collection in some villages. We realized that instead of trying to convince the local stakeholders with the technological aspects and potential impact of the project, we needed to demonstrate the implementation of the data collection in at least one village and present examples of data visualizations that could be used for policy making. The example visualization of dengue fever prevalence shown in Figure 3 is representative of the type of visualization used to convince community leaders of the project’s value.

We learned the importance of engaging community leaders and policymakers at the local level during the preparation phase of the project. This local leadership played an effective role in supporting the research project by facilitating in the selection of potential data collectors, allocation of budget to support data collection, and utilizing the data for policymaking.

#### 4) Data Collection Period

Most data collectors suggested that the period of data collection should be lengthened to allow more time for familiarization with the technological skills required. We had set up 7 months for data collecting process at a very first start of the project, after 6 months passed, we realized that the number of data collectors was not enough. The data collection was also slowed because some data collectors were high schoolers who could only collect data after school hours. In light of this, we had to train new data collectors and extend the data collection period by two months. We learned that when implementing health research using innovative technology, the data collection needed to be sufficient to accommodate un/anticipated delays in resolving technical and human errors.

#### 5) Mobile Device Issues

The mobile devices (smartphones and tablets) used by the data collectors varied widely in terms of operating system (Android or iOS), versions, display sizes, and available features. Technical problems faced by the data collectors were directed to the data technicians at the call centers. Technicians were specialized on either iOS or Android systems, and it was often necessary to fix the same problem multiple times for different types of mobile devices. Because of how difficult it was to anticipate these issues, there was a continuous need for technical expertise throughout the study period. This ensured that compatibility problems could be solved quickly as different types of mobile devices were used for data collection. Future studies would benefit from recruiting developers with expertise in both iOS and Android platforms in order to streamline fixing problems across platforms. There may be a temptation to standardize smartphone/tablet handsets to alleviate many of these issues, and this may be an appropriate solution in some contexts. However, we believe that ultimately the application’s adoption was more robust because of its ability to run on as many different mobile devices as possible.

Some mobile devices simply could not be used for the study as they either did not support 3G SIM cards on the 850MHz frequency or could not locate GPS coordinates.

#### 6) Internet Connectivity Issues

During the implementation phase, the mobile application needed to link the GPS coordinates with the collected data via the internet. This brought about several problems either related to the ability of the mobile device to locate GPS coordinates accurately, the strength of WIFI or 3G signal in the locality or uploading the data into the main system via the internet.

We addressed internet connectivity issues in two ways. First, the development team made changes to the application. The order of questions was revised such that major/important data were collected first. This ensured that if internet connectivity were lost part way through collection, the most essential data would have already been collected. The developers also improved the offline data collection functionality, allowing data to be saved to individual mobile devices and uploaded to the cloud later.

Second, to improve the 3G internet coverage, we coordinated with True Corporation who extended their 3G signals to better cover the study area. We also instructed the data collectors to use other appropriate 3G networks in the area. We found that data service providers were amenable to working with us and suggest that future projects will likely benefit from partnering with local service providers.

#### 7) Geographic Information Availability

We utilized Google Maps Street View as a primary tool in linking data obtained with the questionnaire to geographic locations via GPS coordinates. Street View is a free tool that provides photographs taken by vehicles on-the-ground of all locations along streets. These photographs are collected and uploaded to Google’s database by specialized vehicles equipped with 360-degree cameras and GPS. Street View data were extremely useful in providing precise localization of community members. However, not all of our study area had Street View data available. We reached out to Google and coordinated with them to send Street View cars to expand the available data set in Saraphi district. Google was amenable to the project and expanded its Street View coverage to our entire study area. As with internet connectivity issues, we suggest that future studies should reach out to relevant corporations (i.e. Google) to see if they are willing to help expand coverage in target study areas. For both internet connectivity and geographic information availability, out project would have benefited even more by addressing these issues sooner.

### C. Transferring Lessons of Implementation to New Projects

Over the study period, there were 75 additional provinces throughout Thailand that reached out to us for help adopting the program. 51 provinces successfully completed the training process and were able to develop their community-specific application. Of these 51 provinces, 39 provinces completed at least 60% of their target data collection and are continuing to collect and updated their databases. For these 39 provinces, the application has become a part of their routine health information management with secured funding from local policymakers and stakeholders. The other 12 provinces have all begun some level of data collection.

The ownership of local information and each province’s local version of the application and database system has been transferred to the local government health office. Each province has established its own active committee regarding the maintenance of the project. Long-term maintenance of the project relies very much on the organization-community level.

## IV. Discussion

The present report provided a review of the major challenges that were encountered during the implementation of a community health database system combining mHealth and GIS.

The results of data gathered from focus group discussions, community hearings, questionaries, and targeted interviews indicated that all challenges could be overcome, and a common thread in meeting challenges was the importance of community involvement and constant communication between all involved. Specifically, there were several lessons that should be considered by future teams implementing similar projects.

First, it is important to assess data coverage and geographical data resources early and to partner with companies to expand resources where necessary.

Second, local community leaders should also be approached early in the process. In our project, examples of end products from similar health projects in nearby areas proved most effective in convincing local leaders of the project’s utility.

Third, public awareness of the project should be raised as much as possible. In our project, we created promotional videos and flyers, as well as monthly public hearings

Fourth, data collectors need to be both technologically savvy and have sensitivity and local knowledge when discussing personal issues. In our experience, teaming up younger, tech-savvy data collectors with more-experienced, local data collectors worked best. Alternatively, future projects could allow more time for community training for younger data collectors and technology training for experienced data collectors. It was also important to recruit data collectors from local villages whenever possible.

Fifth, application development is an ongoing process. Projects should plan and budget for a staff of code developers to troubleshoot compatibility and usage issues as they arise throughout.

Sixth, the data collection period should be long enough to allow for setbacks. Ours ended up going two months beyond our initial schedule.

Seventh, continuing engagement between project team members and community stakeholders is critical for catching problems early and improving systems.

Eighth, long term sustainability of public health benefits relies on local leaders and healthcare workers taking ownership and maintenance of the database system and application. This is best accomplished when these stakeholders are involved in the project from the earliest stage possible.

Our experiences were similar to other researchers who have also found that gaining support of community leaders is a common challenge, particularly in rural areas [26], [27]. In Saraphi, we faced low support from the local community at first. Effective community engagement was achieved by increasing dialogue via meetings, a public hearing, and a demonstration of data visualization from a pilot village.

Community engagement yielded unexpected benefits. We gained access to existing public health infrastructure. Feedback from community leaders modified the questionnaire to reflect local needs so data could be more culturally relevant and practically useful to the community.

In the years since the launch of our project in Saraphi district, 75 additional districts across Thailand have at least begun implementing similar projects, and 39 of these have already completed over 60% of their data collection. Across these projects, the keys to success were similar to the lessons learned in Saraphi district. Specifically, team-building strategies to build relationships between project organizers and community members, a budget to fund continuous application development during data collection, and finding community members interested in taking over ownership and maintenance of the project were three critical factors.

In addition to identifying challenges, review of the project’s implementation revealed several unintended benefits. Younger data collectors were given a chance to become more involved in their communities. So far, the large majority of our data collectors have remained active with the program even several years out. Older data collectors have gained new skills in dealing with technology. Saraphi district as a whole has benefited from continued increased 3G and Google Maps Street View coverage.

## V. Conclusion

After required modifications, our GIS health information system was well-optimized for public health use and the lessons learned in this study will serve as a guide for similar public health projects in the future. Our field experiences demonstrate that communication between team members, anticipating technological challenges, and involvement of community members are the three most important areas for future project teams to consider.
